# Imatinib Mesylate Reduces Neurotrophic Factors and pERK and pAKT Expression in Urinary Bladder of Female Mice With Cyclophosphamide-Induced Cystitis

**DOI:** 10.3389/fnsys.2022.884260

**Published:** 2022-04-22

**Authors:** Megan Perkins, Beatrice M. Girard, Susan E. Campbell, Grant W. Hennig, Margaret A. Vizzard

**Affiliations:** ^1^Department of Neurological Sciences, The Larner College of Medicine, The University of Vermont, Burlington, VT, United States; ^2^Department of Pharmacology, The Larner College of Medicine, The University of Vermont, Burlington, VT, United States

**Keywords:** painful bladder syndrome, interstitial cells, platelet-derived growth factor receptor (PDGFR), cytokines, cell signaling, lamina propria

## Abstract

Imatinib mesylate is a tyrosine kinase inhibitor that inhibits platelet-derived growth factor receptor (PDGFR)-α, -β, stem cell factor receptor (c-KIT), and BCR-ABL. PDGFRα is expressed in a subset of interstitial cells in the lamina propria (LP) and detrusor muscle of the urinary bladder. PDGFRα + interstitial cells may contribute to bladder dysfunction conditions such as interstitial cystitis/bladder pain syndrome (IC/BPS) or overactive bladder (OAB). We have previously demonstrated that imatinib prevention via oral gavage or treatment via intravesical infusion improves urinary bladder function in mice with acute (4 hour, h) cyclophosphamide (CYP)-induced cystitis. Here, we investigate potential underlying mechanisms mediating the bladder functional improvement by imatinib using a prevention or treatment experimental design. Using qRT-PCR and ELISAs, we examined inflammatory mediators (NGF, VEGF, BDNF, CCL2, IL-6) previously shown to affect bladder function in CYP-induced cystitis. We also examined the distribution of phosphorylated (p) ERK and pAKT expression in the LP with immunohistochemistry. Imatinib prevention significantly (0.0001 ≤ *p* ≤ 0.05) reduced expression for all mediators examined except NGF, whereas imatinib treatment was without effect. Imatinib prevention and treatment significantly (0.0001 ≤ *p* ≤ 0.05) reduced pERK and pAKT expression in the upper LP (U. LP) and deeper LP (D. LP) in female mice with 4 h CYP-induced cystitis. Although we have previously demonstrated that imatinib prevention or treatment improves bladder function in mice with cystitis, the current studies suggest that reductions in inflammatory mediators contribute to prevention benefits of imatinib but not the treatment benefits of imatinib. Differential effects of imatinib prevention or treatment on inflammatory mediators may be influenced by the route and frequency of imatinib administration and may also suggest other mechanisms (e.g., changes in transepithelial resistance of the urothelium) through which imatinib may affect urinary bladder function following CYP-induced cystitis.

## Introduction

Efficient bladder function is essential for health and high quality of life. Interstitial cystitis/bladder pain syndrome (IC/BPS) is characterized by pelvic pain and/or discomfort, increased urinary urgency and frequency, low volume voids and inflammation (Hanno et al., 2015; Marcu et al., 2018; Akiyama et al., 2020). These functional and inflammatory changes are recapitulated in a cyclophosphamide (CYP)-induced cystitis animal model. CYP treated rodents exhibit decreased time between voiding (i.e., intermicturition intervals, IMIs), decreased infused volume into the bladder to elicit a micturition event (i.e., infused volume, IV), void volume, and increased bladder pressure, afferent nerve excitability and somatic sensitivity (Yoshimura and de Groat, 1999; Boucher et al., 2000; Arms et al., 2013; Gonzalez et al., 2016; Guo et al., 2018) along with altered inflammatory mediator (e.g., growth factors, chemokines, cytokines, and related receptors) expression in the urinary bladder layers and associated dorsal root ganglia (DRG) and spinal cord levels (Malley and Vizzard, 2002; Klinger et al., 2007, 2008; Klinger and Vizzard, 2008; Arms et al., 2010, 2013; Merrill et al., 2012; Gonzalez et al., 2013; Guo et al., 2018).

Receptors associated with inflammation such as transient receptor potential (Trp) channels and the tyrosine kinase receptor, platelet-derived growth factor receptor alpha (PDGFRα), are expressed in a recently identified subset of urinary interstitial cells (Koh et al., 2012; Monaghan et al., 2012; Gevaert et al., 2014b; Heppner et al., 2017; Steiner et al., 2018; Zhao et al., 2021). PDGFRα + is also expressed in multiple tissue (e.g., lung, kidney, intestine, testes, lower urinary tract, CNS) and cell types of mesenchymal origin (e.g., fibroblasts, kidney mesangial cells, astrocytes, platelets, Leydig cells, GI interstitial cells of Cajal) at varying expression throughout development (Heldin and Westermark, 1999; Andrae et al., 2008). Although nomenclature for lower urinary tract (Rothrock et al., 2001) interstitial cells is variable and inconsistent (e.g., fibroblasts, myofibroblasts, telocytes, urinary bladder interstitial cells of Cajal), LUT interstitial cells exhibit distinct morphological and chemical properties (Koh et al., 2012, 2018; Gevaert et al., 2014b,^2017^; Steiner et al., 2018). Recent evidence suggests previous nomenclature refers to two bladder interstitial cell types that differ according to region, chemical expression, and function: (1) Vim+ /PDGFRα+ /CD34-/α-SMA+ /TrpA1+ /desmin-/ANO-1-/c-KIT- in the upper lamina propria (U. LP), and (2) Vim+ /PDGFRα+ /CD34+ /α-SMA-/TrpA1+ /desmin-/ANO-1-/c-KIT- in the deeper lamina propria (D. LP) and detrusor. LP interstitial cells may be involved in the transduction of urinary bladder mechanosensation and detrusor interstitial cells may contribute to regulating detrusor muscle excitability (Wiseman et al., 2003; Ikeda et al., 2007; Gray et al., 2013; Isogai et al., 2016; Heppner et al., 2017; Liu et al., 2017, 2018; Koh et al., 2018; Neuhaus et al., 2020). These studies focus on LP interstitial cells because their potential role as sensory transducers consistent with our research emphasis on sensory signaling mechanisms in LUT function and dysfunction.

Reports indicate altered urinary expression of PDGFRα, Trp channels and other interstitial cell markers in human patients and animal models of bladder dysfunction including IC/BPS, overactive bladder (OAB), spinal cord injury (SCI)-induced OAB, and bladder outlet obstruction (Neuhaus et al., 2005; Mukerji et al., 2006; de Jongh et al., 2007; Ikeda et al., 2007; Kubota et al., 2008; Roosen et al., 2009; Gevaert et al., 2011; Kim et al., 2011; Johnston et al., 2012; Deng et al., 2015; Meng et al., 2015; Preis et al., 2015; Sancho et al., 2017; Liu et al., 2018). Imatinib mesylate is a tyrosine kinase inhibitor that was originally designed to inhibit the BCR-ABL tyrosine kinase in chronic myeloid leukemia, but was also found to inhibit other related receptors, such as PDGFRα (Buchdunger et al., 1996, 2000; Capdeville et al., 2002). Imatinib has been widely used in lower urinary tract (Rothrock et al., 2001) research to disrupt interstitial cell activity and examine the role of interstitial cells in control and pathological bladder (Kubota et al., 2004, 2006; Biers et al., 2006; Sui et al., 2008; Min et al., 2011; Vahabi et al., 2011, 2013; Abrams et al., 2012; Deng et al., 2013; Gevaert et al., 2014a; Kjell et al., 2015; Preis et al., 2015; Sancho et al., 2017; Giglio et al., 2018; Liu et al., 2018). We have recently characterized the urinary bladder functional effects of imatinib administration involving two separate experimental designs (i.e., prevention via oral gavage and treatment via intravesical infusion), in a mouse (female and male) model of CYP-induced cystitis (Perkins et al., 2022). Imatinib administration via gavage or intravesical bladder infusion improved functional (i.e., increased IMI and IV) bladder outcomes in mice with acute (4 hour, h) CYP-induced cystitis. These results suggest that urinary bladder PDGFRα + interstitial cells contribute to the development and/or maintenance of bladder dysfunction. However, imatinib mesylate does inhibit other receptors, in addition to PDGFRα, and off-target effects are possible.

Cytokines, chemokines, growth factors and their related receptors, including PDGFRα, may activate cell signaling pathways such as the MAPK/ERK and PI3K/AKT pathway, which can lead to transcriptional and translational changes promoting cell survival, growth, inflammation, and proliferation (Heldin and Westermark, 1999; Andrae et al., 2008). Increased expression of phosphorylated (p) ERK and pAKT have been reported in the urinary bladder, and lumbosacral DRG and spinal cord of rodents with CYP-induced cystitis, and blockade improves LUT functional outcomes (Cruz et al., 2005; Corrow and Vizzard, 2007, 2009; Qiao and Gulick, 2007; Chung et al., 2010; Arms and Vizzard, 2011; Yu et al., 2012; Coelho et al., 2014; Qiao et al., 2014; Liu et al., 2015; Liang et al., 2016).

Functional improvements with imatinib administration in models of urinary bladder dysfunction, including acute (4 h) CYP-induced cystitis (Perkins et al., 2022; Kubota et al., 2004, 2006; Biers et al., 2006; Abrams et al., 2012; Deng et al., 2013; Kjell et al., 2015; Preis et al., 2015; Sancho et al., 2017; Giglio et al., 2018), may be mediated by multiple factors including changes in the urinary bladder inflammatory milieu and/or cell signaling pathways. The effect of imatinib on the inflammatory milieu and signaling pathways in animal models of IC/BPS has not been previously studied; however, the anti-inflammatory properties of imatinib have been shown in other systems (Capdeville et al., 2002; Ma et al., 2011; Abrams et al., 2012; Hayashi et al., 2012; Adzemovic et al., 2013; Kjell et al., 2015). In this study, we examine the effect of imatinib administration using a prevention design, via oral gavage, or treatment design, via intravesical bladder infusion, on urinary inflammatory mediators and cell signaling kinases in an acute (4 h) CYP-induced cystitis female mouse model, using real time quantitative reverse transcription-polymerase chain reaction (qRT-PCR), enzyme-linked immunosorbent assays (ELISAs), and immunohistochemistry (IHC). We examined the inflammatory mediators, BDNF, NGF, VEGF, IL-6, and CCL2, and signaling kinases, pERK and pAKT, in the urinary bladder and LP, due to their established involvement in CYP-induced cystitis (Vizzard, 2000b; Malley and Vizzard, 2002; Murray et al., 2004; Corrow and Vizzard, 2007, 2009; Arms and Vizzard, 2011; Girard et al., 2011a,^2016^; Arms et al., 2013).

## Materials and Methods

### Experimental Animals

Female wildtype (WT), C57BL/6 mice (3–5 months) (Jackson Labs, Bar Harbor, ME, United States) were used in these experiments. The UVM Institutional Animal Care and Use Committee (IACUC) approved all experimental procedures involving animal use (IACUC #X9-020). Mice were bred and housed in standard laboratory conditions (Girard et al., 2016; Guo et al., 2018; Tooke et al., 2019) at The Larner College of Medicine. University of Vermont (UVM) animal facilities and the UVM Office of Animal Care and Management supervised animal care according to the American Association for Accreditation of Laboratory Animal Care (AAALAC) and NIH guidelines. Efforts were taken to minimize animal pain and distress. Mice demonstrating signs of pain and distress that could not be mitigated with post-operative analgesics were immediately euthanized. Separate cohorts of littermate mice were used. Only female mice were used due to the increased prevalence of IC/BPS in human females (Hanno et al., 2015). The estrous cycle status of mice was not determined.

### Cyclophosphamide Dosing and Administration

Acute cystitis was modeled with a single CYP injection (200 mg/kg, i.p.) (Malley and Vizzard, 2002; Bjorling et al., 2011; Guo et al., 2018). Mice were anesthetized (5% isoflurane in oxygen), euthanized by thoracotomy, and urinary bladders were collected for qRT-PCR, ELISAs or IHC experiments (as detailed below) 4 h post-CYP injection. Control cohorts received no treatment. We have previously shown no functional differences or differences in neuroactive compound expression in LUT for rodents treated with CYP vehicle (sterile water) or rodents receiving no vehicle (Vizzard, 2000a,c).

### Imatinib Mesylate Dosing and Administration

**FIGURE 1:**
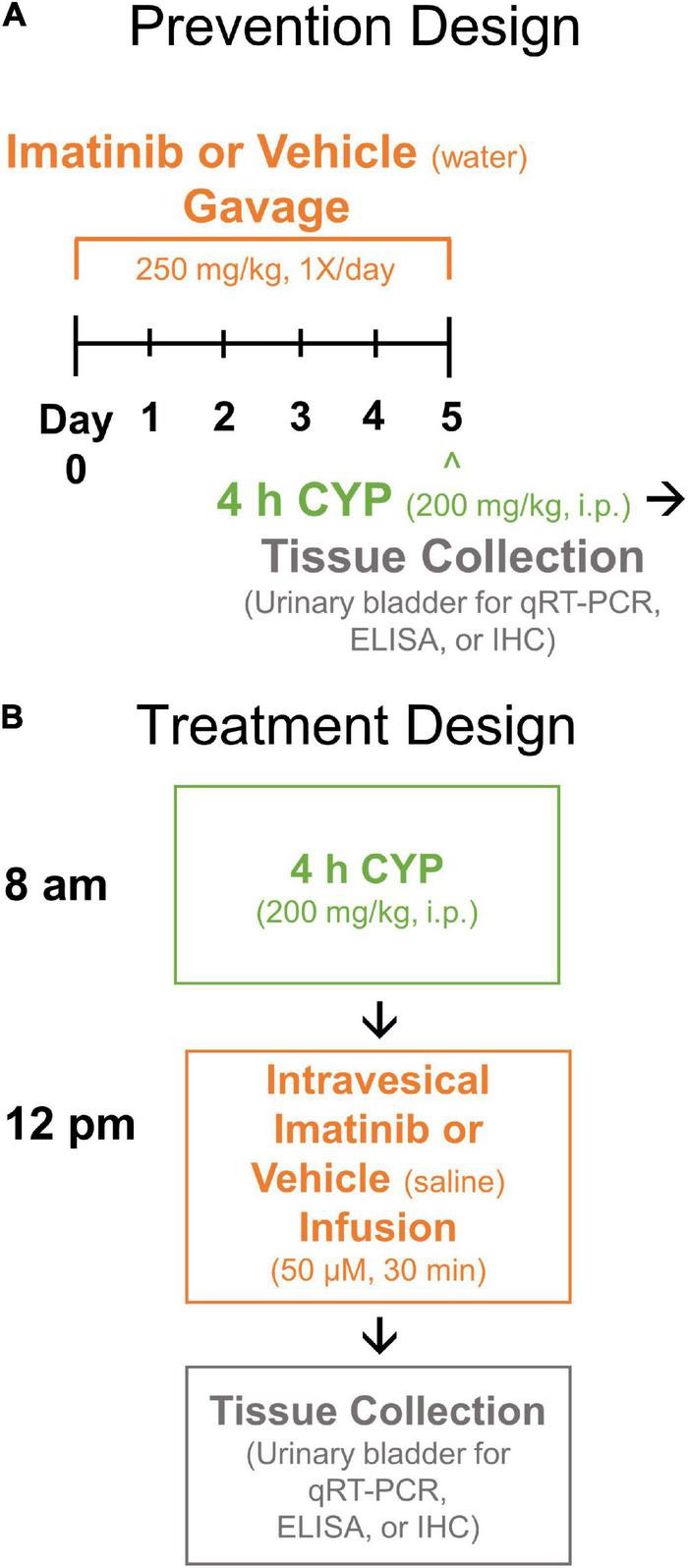
Experimental designs. **(A)** Prevention design. Mice were pre-treated with imatinib mesylate (250 mg/kg) or vehicle (water) via oral gavage for 5 days, 1X/day. Mice received acute (4 h) cyclophosphamide (CYP) treatment (200 mg/kg, i.p.) on the 5th day. **(B)** Treatment design. Mice received acute (4 h) CYP (200 mg/kg, i.p.) followed 4 h later by imatinib (50 μM) or vehicle (saline) treatment via transurethral intravesical (intrabladder) infusion (30 min). Upon completion of prevention or treatment design, urinary bladders were collected (4 h + post CYP) for real time qRT-PCR, ELISA or IHC experiments. qRT-PCR, quantitative reverse transcription-polymerase chain reaction; ELISA, enzyme-linked immunosorbent assay; IHC, immunohistochemistry.

We determined the dose and route of imatinib (LC Laboratories, Woburn, MA, United States) administration in previous pilot studies (data not shown) with the guidance of previous reports (Abrams et al., 2012; Kjell et al., 2015; Sancho et al., 2017). Two different delivery routes and schedules were used in these experiments: (1) systemically via oral gavage (250 mg/kg; 22 gauge/25 mm, stainless steel) (Figure 1A); or (2) directly via intravesical (i.e., intrabladder) infusion (50 μM) with a transurethral catheter (Figure 1B). Mice were euthanized (5% isoflurane in oxygen; thoracotomy) for urinary bladder collection after imatinib or vehicle (i.e., saline or water), and CYP administration depending on schedules (Figure 1).

#### Oral Gavage (Prevention Schedule)

Mice were placed into a small polyethylene, cone-shaped device with an opening at the far end, allowing for proper mouse placement (180° upright, mouth oriented upward) for oral gavage. Mice were constantly monitored to ensure correct gavage placement and animal safety. To maintain consistency, administration was performed in the morning (8 am-12 pm) by the same individual each day.

Before acute cystitis induction with CYP (4 h, 200 mg/kg, i.p.) mice were pre-treated with imatinib (250 mg/kg, 1X/day) or vehicle (water) for 5 days. On the 5th (last) experimental day, mice received imatinib via gavage and were left undisturbed in their home cage for 30 min prior to CYP administration (Figure 1A).

#### Transurethral Intravesical Infusion (Treatment Schedule)

Four (4) hour post-CYP administration (200 mg/kg, i.p.), mice were anesthetized and placed supine on a water mat heating pad. Breathing rate was monitored while mice were anesthetized to ensure mouse well-being. Bladders were manually expressed before catheterization. Lubricated polyethylene tubing (PE-10, Clay Adams, Parsippany, NJ, United States) was inserted through the urethra to infuse imatinib (50 μM) or vehicle (saline) into the bladder, until a full bladder (cystitis, 25–100 μL; control, 100–400 μL) could be palpated. The catheter was then removed, and mice remained anesthetized for 30 min (Figure 1B).

### Immunohistochemistry

Whole, urinary bladders were dissected from female, WT mice after (1) imatinib prevention (oral gavage) or (2) treatment (transurethral intravesical infusion), with or without 4 h CYP. IHC was performed as previously described (Arms et al., 2013; Girard et al., 2016; Guo et al., 2018). Bladders were conditioned (2% paraformaldehyde + 0.02% picric acid, and 30% sucrose) and embedded in OCT (optimal cutting temperature compound). Three (3)-8 bladder sections (10 μm) were used for IHC and were randomly selected from all regions of the urinary bladder with the LP present. Primary and secondary antibodies are listed in Table 1. Slides were mounted with 1 μL DAPI (Sigma-Aldrich, St. Louis, MO, United States)/1 mL CitiFluor AF2 (Electron Microscopy Sciences, Hatfield, PA, United States). Methodological and procedural controls included: incubation of tissue with (1) primary antibody but without secondary antibody, (2) secondary antibody but without primary antibody, and (3) blocking solution without primary or secondary antibodies. All tissues (treatment and controls) were processed simultaneously.

**TABLE 1:** Antibodies used for immunohistochemistry.

Primary antibody	Dilution	Source	Secondary antibody	Dilution	Source
Rabbit anti-pPDGFRα	1:250	Abcam, Cambridge, United Kingdom	FITC donkey anti-rabbit	1:200	Jackson Immunoresearch, West Grove, PA, United States
Sheep anti-pERK	1:1000	Osenses, Keswick, SA, Australia	CY3 donkey anti-sheep	1:500	Jackson Immunoresearch
Sheep anti-pAKT	1:3000	Osenses	CY3 donkey anti-sheep	1:500	Jackson Immunoresearch

*pPDGFRα, phosphorylated platelet-derived growth factor receptor alpha; pERK, phosphorylated extracellular signal-related kinase; pAKT, phosphorylated protein kinase B; FITC, fluorescein isothiocyanate; CY3, sulfo-cyanine3.*

### Visualization and Semi-Quantitative Analysis of Lamina Propria Immunoreactivity

Bladder sections were imaged using a Yokogawa CSU-W1 confocal, spinning disk system coupled to a Nikon Eclipse NI-E microscope (Microscopy Imaging Center, UVM). Bladder sections were simultaneously imaged beginning with the brightest condition (4 h CYP groups). FITC staining was visualized with a filter excitation range of 488 nm and emission range of 525 nm. CY3 staining was visualized with a filter excitation range of 560–596 nm and emission range of 610–655 nm. Images were saved and exported as ND2 files. In the urinary bladder, upper LP (U. LP) and deeper LP (D. LP) regions were isolated individually by thresholding until the entire region was selected (FIJI Image Analysis Software) (Schindelin et al., 2012). The pPDGFRα antibody acted as a tissue orientation reference, which distinctly defined the U. LP and D. LP. Binary gray-scaled (8-bit) masks were then produced of either the U. LP (i.e., fibrous band of tissue immediately deep to the urothelium) or D. LP (i.e., loosely distributed region of tissue between the upper LP and detrusor). The mask and images for both channels (CY3, FITC) were saved as TIFFs and imported into the image analysis software, Volumetry (VolumetryG9a, Grant Hennig) (Cobine et al., 2010). A combination image was created from the red and green channel images, with either the U. LP or D. LP mask overlayed. FITC and CY3 fluorescence intensity were measured (VolumetryG9a, Grant Hennig) (Cobine et al., 2010). Exported data included pixel intensity, number of pixels fluorescing/each intensity level, and the total area measured. The number of pixels fluorescing at a particular intensity level was divided by the total measured area. These data were then displayed as a histogram with pixel intensity (intensity unit, IU_8–bit_) (*x*-axis) and pixel number or frequency/intensity measurement (*y*-axis), producing intensity curves. Intensity curves were created for 2–6 sections/mouse and 5–8 mice/group. The intensity curves from multiple sections from the same mouse were averaged/mouse. Once intensity curves were determined for each mouse within a group, curves were averaged/group. The median of each group’s fluorescence curve, which generally corresponded to the peak of the curve, was compared using a two-way ANOVA and Bonferroni’s multiple comparisons test.

### Immunohistochemistry Figure Preparation

Digital images were captured with two Andor Ixon EM CCD cameras coupled to a Yokogawa CSU-W1 spinning disk confocal system and microscope. Imaging settings were held constant for all image acquisition. Images were assembled and labeled using Microsoft Paint (Windows 10, version 20H2, Microsoft Corporation ©).

### Lamina Propria Preparation for Quantitative Reverse Transcription-Polymerase Chain Reaction

Whole, urinary bladders were dissected from female, WT mice after (1) imatinib prevention (oral gavage) or (2) treatment (transurethral intravesical infusion), with or without 4 h CYP. Bladders were immediately transferred to a modified HEPES solution (10 mM HEPES, 6 mM KCl, 134 mM NaCl, 7 mM glucose, pH = 7.4). The LP was separated from the detrusor and urothelium using fine tip forceps under a dissecting microscope as previously described (Girard et al., 2016; Guo et al., 2018). The LP was stored at –80°C and the excess tissue discarded.

### Real Time Quantitative Reverse Transcription-Polymerase Chain Reaction

Real time qRT-PCR was used to determine mRNA transcript expression in the LP of the urinary bladder for multiple inflammatory mediators (i.e., BDNF, NGF, VEGFaa, VEGFab, IL-6, CCL2), as previously described (Girard et al., 2011a,b, 2016; Tooke et al., 2019). Total RNA was extracted using the Stat-60 total RNA/mRNA isolation procedure (Tel-Test “B,” Friendswood, TX, United States). To reverse transcribe complementary DNA (cDNA), 1 μg of RNA/sample was mixed with oligo dT primers, random hexamers, M-MLV buffer, M-MLV reverse transcriptase (Promega Corp., Madison, WI, United States), dNTP, RNasin^®^ ribonuclease inhibitor and molecular grade water, producing a 25 μL final reaction volume. To establish quantitative PCR standards for all transcripts, the amplified cDNA was ligated directly to the pCR2.1 TOPO vector, using the TOPO TA cloning kit (Invitrogen, Carlsbad, CA, United States). Nucleotide sequences were verified by automated fluorescence dideoxy die terminator sequencing (Vermont Cancer Center DNA Analysis Facility). Ten-fold serial dilutions were prepared for each stock plasmid to establish quantitative standards and estimate the relative expression of the receptor transcripts. cDNA templates were diluted 10-fold to reduce inhibitory effects of the reverse transcription reaction components. cDNA dilutions were assayed using Luna^®^ Universal qPCR Master Mix (New England Biolabs, Ipswich, MA, United States) and 300 nM from each primer, producing a total reaction volume of 20 μL. The Applied Biosystems 7500 Fast real-time PCR system (Applied Biosystems, Foster City, CA, United States) was used for real-time PCR. The following standard conditions were applied: (1) serial heating at 94°C for 2 min and (2) amplification over 45 cycles at 94°C for 15 s at 60–64°C depending on primers set for 30 s. The amplified product underwent SYBR Green I melting analysis. A single DNA melting profile was observed under these dissociation assay conditions, demonstrating the amplification of a single unique product free of primer dimers or other anomalous products. Oligonucleotide primer sequences are listed in Table 2.

**TABLE 2:** Primer sequences for real time qRT-PCR.

Primer	Sequence
BDNF	U: GTGACAGTATTAGCGAGTGGG L: GGGTAGTTCGGCATTGC
NGF	U: AGTGAGGTGCATAGCGTAAT L: AGTGGAGTCTCCGTTTCTTA
VEGFaa	U: AGAGCGGAGAAAGCATTTGTTT L: AGCATTTGTTTGTCCAAGATCC
VEGFab	U: TCACCGCCTTGGCTTGTCACAT L: TTAATCGGTCTTTCCTTTGAGA
IL-6	U: TTGCCTTCTTGGGACTGATG L: GCCATTGCACAACTCTTTTC
CCL2	U: AGCCAACTCTCACTGAAGC L: GTGAATGAGTAGCAGCAGGT
L32	U: CCTGGCGTTGGGATTGGTGA L: GAAAAGCCATCGTAGAAAGA

*BDNF, brain-derived neurotrophic factor; NGF, nerve growth factor; VEGFaa, vascular endothelial growth factor aa; VEGFab, vascular endothelial growth factor ab; IL-6, interleukin 6; CCL2, chemokine (C-C motif) ligand 2; L32, ribosomal protein L32. U: upper; L: lower.*

### Data Analysis for mRNA Quantification

Quantification of the mRNA transcripts was analyzed as previously described (Gonzalez et al., 2013; Girard et al., 2016; Tooke et al., 2019). All data are expressed as the relative quantity of the gene of interest, normalized to the relative quantity of the housekeeping gene L32. All data are displayed as fold change with respect to the control (vehicle, no CYP) group.

### Enzyme Linked Immunosorbent Assays for Protein Quantification

Whole, urinary bladders were dissected from the female, WT mice that received either imatinib prevention (gavage) or treatment (intravesical), with or without acute (4 h) CYP treatment. Bladders were weighed and placed in collection tubes with Tissue Protein Extraction Reagent (1 g tissue/20 mL) with complete protease inhibitor cocktail tablets (Roche Applied Science, Mannheim, Germany) and stored at –20°C. All protein samples were assayed simultaneously. Enzyme linked immunosorbent assays (ELISA) (i.e., NGF, BDNF, VEGF, IL-6, CCL2) and Bradford assays were performed as previously described (Arms et al., 2013; Gonzalez et al., 2013; Girard et al., 2016, 2021; Guo et al., 2018) and according to the manufacturer’s instructions (DuoSet^®^ ELISA Development Systems, R&D Systems, Minneapolis, MN, United States).

### Enzyme Linked Immunosorbent Assay Analysis for Protein Quantification

The standards provided with this protocol generated a linear standard curve for each mediator measured. The background absorbance was subtracted from the samples and standards absorbance values. Most ELISA samples were run without dilution although some ELISA samples (CCL2; imatinib infusion samples) required further dilution. BDNF and CCL2 ELISA samples for the imatinib pre-treated (gavage) samples exceeded the upper limits of the standard curve (BDNF: 3358 pg/mL, CCL2: 364 pg/mL). Unfortunately, we did not have sufficient remaining tissue samples to repeat these ELISAs with appropriate dilution. The curve fitting of the sample protein content values to standard values was estimated using a least-squares fit analysis as previously described (Arms et al., 2013; Gonzalez et al., 2013; Girard et al., 2016, 2021; Guo et al., 2018).

### Statistical Analyses

All values represent fold change ± SEM with respect to vehicle control. Data were analyzed using a two-way analysis of variance (ANOVA). When the *F* ratio exceeded the critical value at α = 0.05, Tukey’s multiple comparisons test was performed. Analyses were performed on raw data values before converting to fold change. All analyses were performed using GraphPad Prism software (GraphPad Prism version 8.0.0 for Windows, GraphPad Software, San Diego, CA, United States^1^).

## Results

### Oral Gavage of Imatinib Reduces IL-6, CCL2, Brain-Derived Neurotrophic Factor and Vascular Endothelial Growth Factor, but Not Nerve Growth Factor Lamina Propria mRNA and Reduces IL-6 and Vascular Endothelial Growth Factor Protein (Whole Bladder) Expression in Female Mice With 4 h Cyclophosphamide-Induced Cystitis

#### mRNA Expression

**FIGURE 2:**
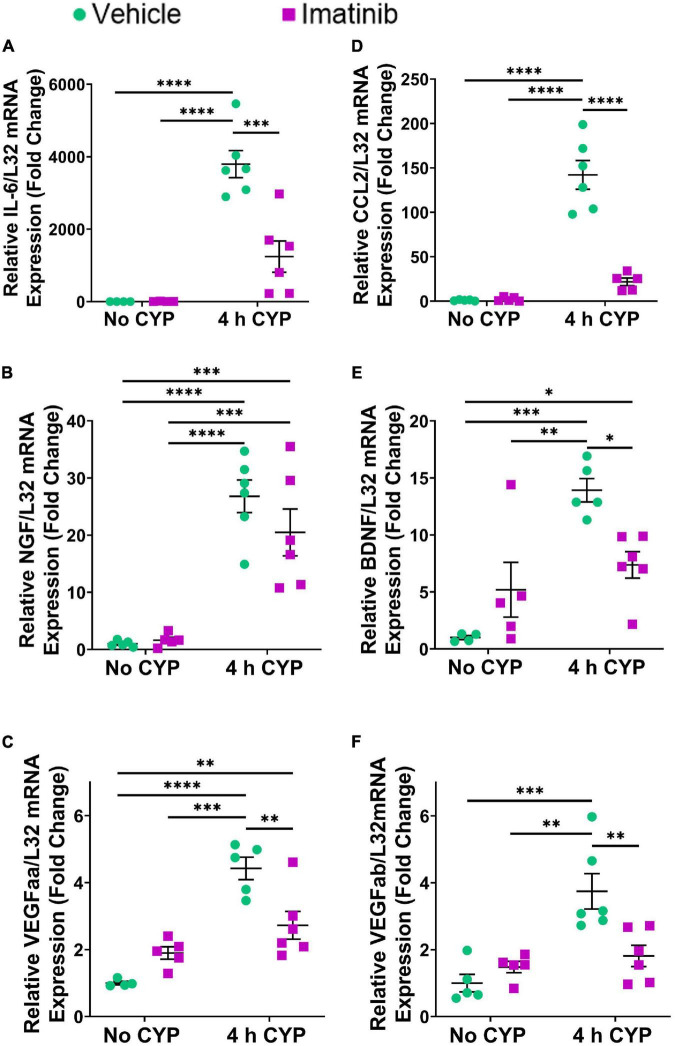
Oral gavage of imatinib mesylate significantly affects inflammatory mediator mRNA transcript expression in the lamina propria (LP) of the urinary bladder in an acute (4 h) cyclophosphamide (CYP)-induced cystitis female mouse model. Acute (4 h) CYP treatment (200 mg/kg, i.p.) significantly (0.0001 ≤ *p* ≤ 0.001) increased **(A)** IL-6 (3794.4-fold), **(B)** NGF (26.8-fold), **(C)** VEGFaa (4.4-fold), **(D)** CCL2 (142.2-fold), **(E)** BDNF (13.9-fold), and **(F)** VEGFab (3.7-fold) mRNA transcript expression in the bladder LP in female mice pre-treated with vehicle (water) via gavage, compared to vehicle (water gavage, no CYP) control. Imatinib prevention (250 mg/kg, 5 days, 1X/day) via oral gavage significantly (0.0001 ≤ *p* ≤ 0.05) decreased **(A)** IL-6 (3.1-fold), **(C)** VEGFaa (1.6-fold), **(D)** CCL2 (6.5-fold), **(E)** BDNF (1.9-fold), and **(F)** VEGFab (2.1-fold), but did not affect **(B)** NGF, mRNA transcript expression in the bladder LP of female mice with 4 h CYP-induced cystitis. mRNA transcripts were measured by real time qRT-PCR. mRNA transcript expression was normalized to expression of the house keeping gene, L32. Transcript values are displayed as fold change values ± SEM with respect to the control group (vehicle, no CYP). *n* = 4–6. *p ≤ 0.05; **p ≤ 0.01; ***p ≤ 0.001; ****p ≤ 0.0001 by two-way ANOVA with Tukey’s multiple comparisons test.

Acute (4 h) CYP treatment (200 mg/kg, i.p.) significantly (*p* ≤ 0.01) increased IL-6, CCL2, NGF, BDNF and VEGF isoforms, VEGFaa and VEGFab LP mRNA expression, compared to control (no CYP) and imatinib (no CYP) groups (3.7–3794.4-fold) (Figures 2A–F). Imatinib prevention by oral gavage (250 mg/kg) significantly (*p* ≤ 0.05) decreased VEGFaa, VEGFab, IL-6, CCL2 and BDNF LP mRNA but did not affect NGF LP mRNA in mice with 4 h CYP-induced cystitis (1.6–6.5-fold) (Figures 2A–F). mRNA expression was not significantly different in mice with 4 h CYP-induced cystitis pre-treated with imatinib, compared to control (no CYP) groups, except for VEGFaa and BDNF (Figures 2C,E).

#### Protein Expression

**FIGURE 3:**
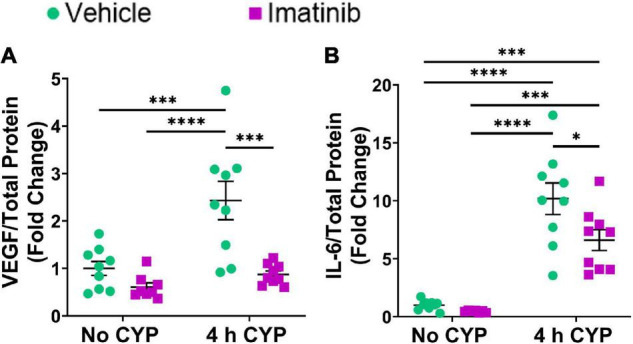
Oral gavage of imatinib mesylate significantly decreases inflammatory mediator protein expression the urinary bladder in an acute (4 h) cyclophosphamide (CYP)-induced cystitis female mouse model. Acute (4 h) CYP treatment (200 mg/kg, i.p.) significantly (0.0001 ≤ *p* ≤ 0.001) increased **(A)** VEGF (2.4-fold) and **(B)** IL-6 (10.2-fold) whole bladder protein expression in female mice pre-treated with vehicle (water) via gavage, compared to vehicle (water gavage, no CYP) control. Imatinib prevention (250 mg/kg, 5 days, 1X/day) via oral gavage significantly (0.001 ≤ *p* ≤ 0.05) decreased **(A)** VEGF (2.8-fold) and **(B)** IL-6 (1.5-fold) whole bladder protein expression in female mice with 4 h CYP-induced cystitis. Sample values for CCL2 and BDNF ELISAs, from the prevention design studies, exceeded the upper limits of the standard curves and assays. We did not have sufficient remaining tissue samples to repeat these ELISAs with appropriate dilution. Raw ELISA values (pg/mL) are presented as fold change values ± SEM with respect to the control group (vehicle, no CYP). *n* = 7–9. *p ≤ 0.05; **p ≤ 0.01; ***p ≤ 0.001; ****p ≤ 0.0001 by two-way ANOVA with Tukey’s multiple comparisons test.

Acute (4 h) CYP treatment significantly (*p* ≤ 0.001) increased VEGF and IL-6 protein expression compared to vehicle and imatinib controls (2.4–10.2-fold) (Figure 3). Imatinib prevention by oral gavage (250 mg/kg) significantly (*p* ≤ 0.05) decreased VEGF and IL-6 protein expression in 4 h CYP-induced cystitis mice (1.6–2.8-fold). VEGF protein expression was reduced to control levels in acute (4 h) CYP-induced cystitis mice pre-treated with imatinib (Figure 3A). Although imatinib prevention significantly (*p* ≤ 0.05) reduced IL-6 expression in the acute CYP condition, expression remained significantly (*p* ≤ 0.001) increased compared to vehicle and imatinib controls (6.6–14.4-fold) (Figure 3B). ELISAs for CCL2 and BDNF were also conducted; however, sample values exceeded the upper limits of the standard curves and assays could not be reported because of limited protein yield.

### Transurethral Bladder Infusion of Imatinib Does Not Reduce Inflammatory Mediator mRNA (Lamina Propria) and Protein (Whole Bladder) Expression in Female Mice With 4 h Cyclophosphamide-Induced Cystitis

**FIGURE 4:**
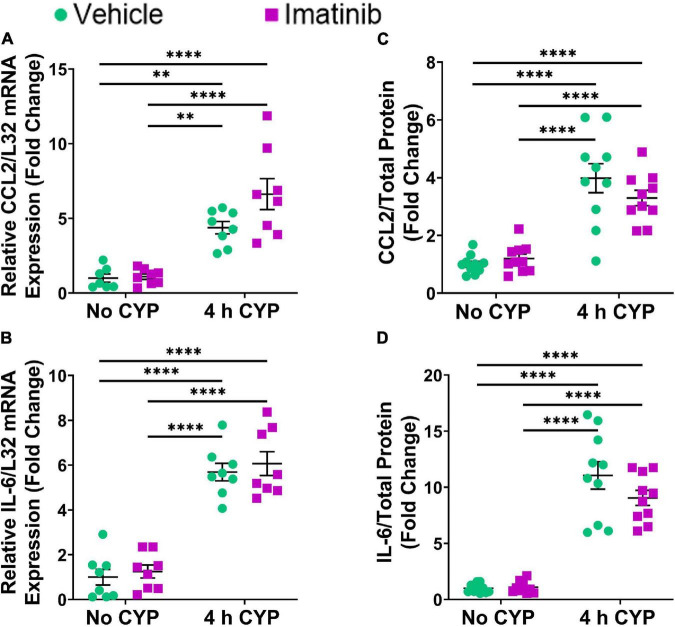
Transurethral bladder infusion of imatinib mesylate does not significantly affect CCL2 and IL-6 mRNA transcript (lamina propria, LP) or protein (whole urinary bladder) expression in an acute (4 h) cyclophosphamide (CYP)-induced cystitis female mouse model. Acute (4 h) CYP treatment (200 mg/kg, i.p.) significantly (0.0001 ≤ *p* ≤ 0.01) increased **(A)** CCL2 (4.4-fold) and **(B)** IL-6 (5.7-fold) mRNA transcript expression in the bladder LP, and **(C)** CCL2 (4-fold) and **(D)** IL-6 (11.1-fold) whole bladder protein expression in female mice that received vehicle (saline) intravesical infusion 4 h post CYP injection, compared to vehicle infused control. Imatinib treatment via transurethral intravesical infusion (50 μM, 30 min) 4 h post CYP administration, did not significantly affect **(A)** CCL2 or **(B)** IL-6 mRNA transcript expression in the bladder LP, nor **(C)** CCL2 or **(D)** IL-6 protein expression in whole bladder samples in female mice with 4 h CYP-induced cystitis. mRNA transcript and protein expression were measured by real time qRT-PCR and ELISAs, respectively. mRNA transcript expression was normalized to expression of the house keeping gene, L32. Raw mRNA transcript and ELISA values (pg/mL) are presented as fold change values ± SEM with respect to the control group (vehicle, no CYP). *n* = 7–10. *p ≤ 0.05; **p ≤ 0.01; ***p ≤ 0.001; ****p ≤ 0.0001 by two-way ANOVA with Tukey’s multiple comparisons test.

**FIGURE 5:**
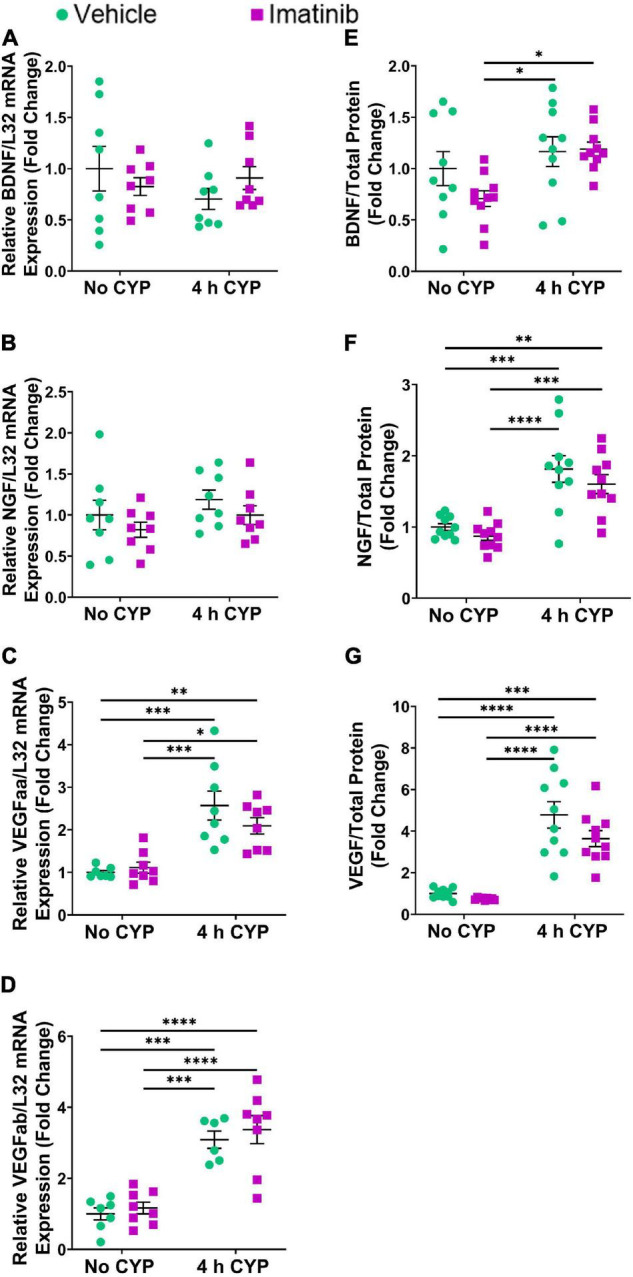
Transurethral bladder infusion of imatinib mesylate does not significantly affect VEGF, BDNF or NGF mRNA transcript (lamina propria, LP) or protein (whole urinary bladder) expression in an acute (4 h) cyclophosphamide (CYP)-induced cystitis female mouse model. Acute (4 h) CYP treatment (200 mg/kg, i.p.) significantly (*p* ≤ 0.001) increased **(C)** VEGFaa (2.6-fold) and **(D)** VEGFab (3.1-fold) mRNA but did not affect **(A)** BDNF or **(B)** NGF mRNA transcript expression in the bladder LP, in female mice that received vehicle (saline) intravesical infusion 4 h post CYP injection, compared to vehicle infused (no CYP) controls. 4 h CYP treatment significantly (0.0001 ≤ *p* ≤ 0.001) increased **(F)** NGF (1.8-fold) and **(G)** VEGF (4.8-fold) but did not affect **(E)** BDNF whole bladder protein expression, compared to vehicle controls. Imatinib treatment via transurethral intravesical infusion (50 μM, 30 min) 4 h post CYP administration, did not significantly affect **(A)** BDNF, **(B)** NGF, **(C)** VEGFaa, or **(D)** VEGFab mRNA transcript expression in the bladder LP, nor **(E)** BDNF, **(F)** NGF, or **(G)** VEGF protein expression in whole bladder samples in female mice with 4 h CYP-induced cystitis. mRNA transcript and protein expression were measured by real time qRT-PCR and ELISAs, respectively. mRNA transcript expression was normalized to expression of the house keeping gene, L32. Raw mRNA transcript and ELISA values (pg/mL) are presented as fold change values ± SEM with respect to the control group (vehicle, no CYP). *n* = 6–10. *p ≤ 0.05; **p ≤ 0.01; ***p ≤ 0.001; ****p ≤ 0.0001 by two-way ANOVA with Tukey’s multiple comparisons test.

Acute (4 h) CYP (200 mg/kg, i.p.) treatment significantly (*p* ≤ 0.01) increased CCL2, IL-6, and VEGF isoforms mRNA (2.3–5.7-fold) (Figures 4A,B, 5C,D) and CCL2, IL-6, NGF, and VEGF protein expression (1.8–11.1-fold) (Figures 4C,D, 5F,G) in the LP and urinary bladder, respectively, in mice that received saline intrabladder infusion. However, 4 h CYP treatment did not affect BDNF or NGF LP mRNA expression (Figures 5A,B). BDNF urinary bladder protein expression was significantly (*p* ≤ 0.05) increased in mice with 4 h CYP-induced cystitis that received saline or imatinib (1.7-fold each) intrabladder infusion, compared to imatinib infused controls (no CYP), but was not significantly different from vehicle (saline) infused controls (Figure 5E). Imatinib treatment via transurethral bladder infusion did not significantly affect CCL2, IL-6, BDNF, NGF, VEGFaa or VEGFab LP mRNA or urinary bladder protein expression in female mice with 4 h CYP cystitis (Figures 4, 5).

### Oral Gavage, but Not Transurethral Bladder Infusion, of Imatinib Reduces Bladder Weight in Female Mice With 4 h Cyclophosphamide-Induced Cystitis

**FIGURE 6:**
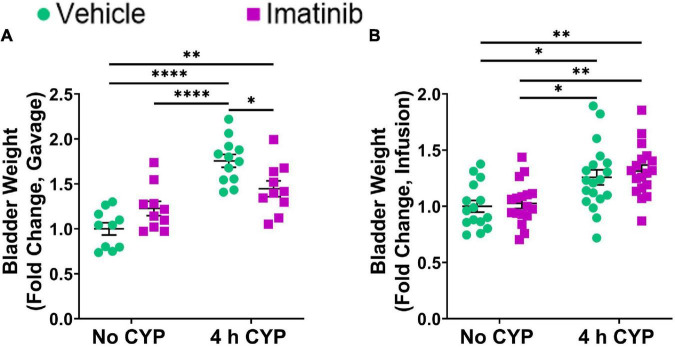
Oral gavage of imatinib mesylate, but not transurethral bladder infusion, significantly reduces bladder weight in an acute (4 h) cyclophosphamide (CYP)-induced cystitis female mouse model. Acute (4 h) CYP treatment (200 mg/kg, i.p.) significantly (0.0001 ≤ *p* ≤ 0.05) increased bladder weight in **(A)** mice that received vehicle (water) via oral gavage for 5 days prior to CYP administration (1.8-fold) and **(B)** mice that received transurethral vehicle (saline) intravesical infusion 4 h post CYP administration (1.3-fold), compared to vehicle (no CYP) controls. **(A)** Imatinib prevention (250 mg/kg, 5 days, 1X/day) via oral gavage significantly (*p* ≤ 0.05) (1.2-fold) reduced bladder weight in female mice with 4 h CYP-induced cystitis, but **(B)** imatinib treatment (50 μM, 30 min) via intravesical infusion did not affect bladder weight. Raw values (g) are presented as fold change values ± SEM with respect to the control group (vehicle, no CYP). *n* = 10–19. *p ≤ 0.05; **p ≤ 0.01; ***p ≤ 0.001; ****p ≤ 0.0001 by two-way ANOVA with Tukey’s multiple comparisons test.

Four hour CYP treatment significantly (*p* ≤ 0.05) increased bladder weight compared to vehicle and imatinib controls (1.2–1.8-fold) (Figure 6). Imatinib prevention significantly (*p* ≤ 0.05) (1.2-fold) reduced bladder weight in mice with 4 h CYP-induced cystitis (Figure 6A) whereas, imatinib treatment was without effect (Figure 6B).

### Oral Gavage or Transurethral Bladder Infusion of Imatinib Reduces pERK and pAKT Expression in the Upper and Deeper Lamina Propria in Mice With 4 h Cyclophosphamide-Induced Cystitis

**FIGURE 7:**
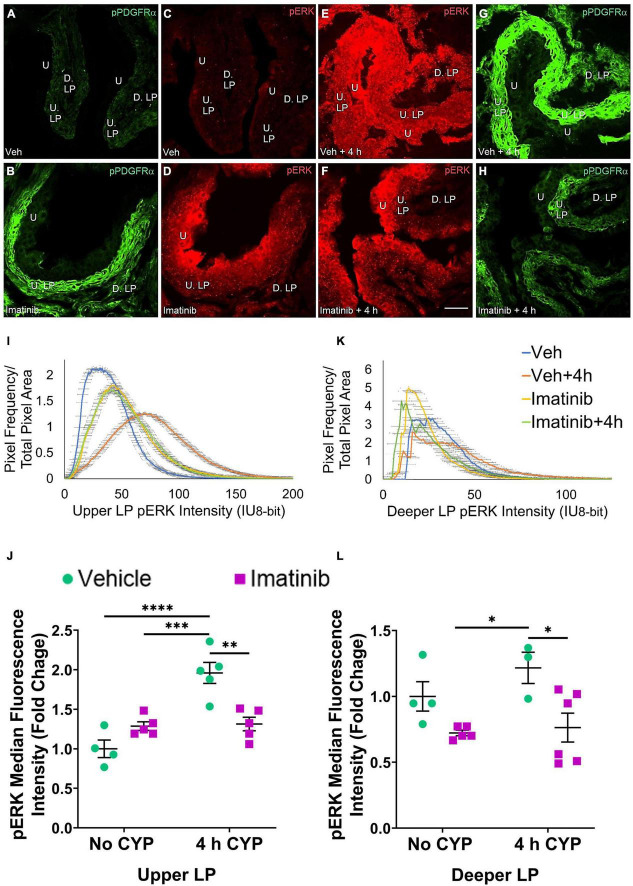
Oral gavage of imatinib mesylate reduces pERK expression in the upper lamina propria (U. LP) and deeper lamina propria (D. LP), in an acute (4 h) cyclophosphamide (CYP)-induced cystitis female mouse model. Representative **(A–H)** images, **(I)** upper LP and **(K)** deeper LP fluorescence intensity curves of pERK immunoreactivity (CY3, red) in the urinary bladder (10 μm sections) in female mice with **(E)** acute (4 h) cyclophosphamide (CYP)-induced cystitis (200 mg/kg, i.p.) and **(C,D)** controls (no CYP), **(F)** pre-treated with imatinib mesylate via oral gavage (250 mg/kg, 5 days, 1X/day). **(A,B,G,H)** Bladder tissue was co-stained for pPDGFRα (FITC, green), and the corresponding images are displayed to provide orientation. 4 h CYP treatment significantly (*p* ≤ 0.0001) increased pERK expression in the **(J)** U. LP (2-fold) but not the **(L)** D. LP, compared to vehicle controls. Imatinib prevention significantly (0.01 ≤ *p* ≤ 0.05) reduced pERK expression in the **(J)** U. LP (1.5-fold) and the **(L)** D. LP (1.6-fold) in female mice with 4 h CYP-induced cystitis. Raw IHC values (intensity units, 8-bit) are presented as fold change values ± SEM with respect to the control group (vehicle, no CYP). *n* = 3–6. *p ≤ 0.05; **p ≤ 0.01; ***p ≤ 0.001; ****p ≤ 0.0001 by two-way ANOVA with Tukey’s multiple comparisons test. Tissues were processed simultaneously. Microscope and camera settings were kept constant across all groups. Fluorescence intensity was measured using Volumetry image analysis software (VolumetryG9a, Grant Hennig). 10 μm sections. Scale bar = 50 μm. U, urothelium; U. LP, upper lamina propria; D. LP, deeper lamina propria; IU, intensity units.

**FIGURE 8:**
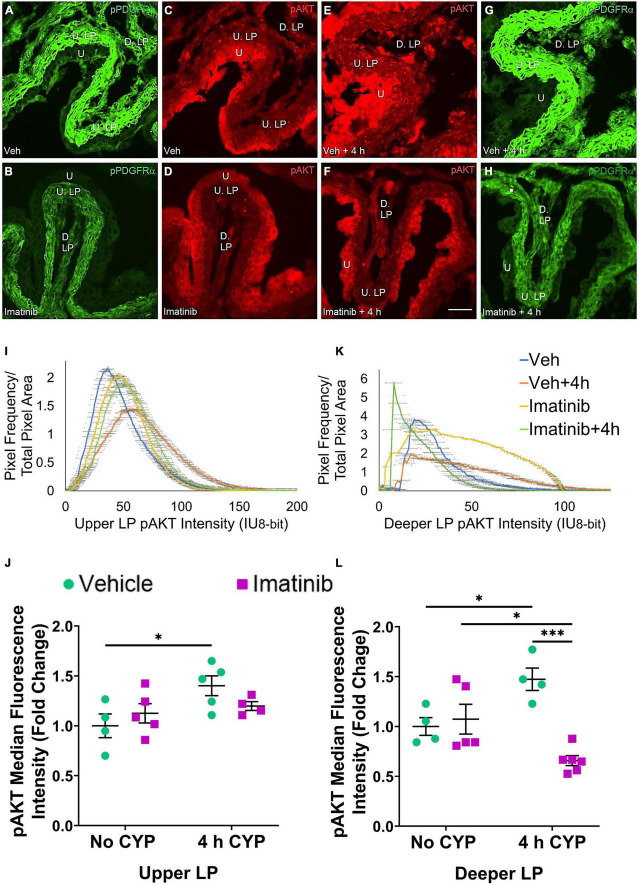
Oral gavage of imatinib mesylate reduces pAKT expression in the deeper lamina propria (D. LP), but not upper lamina propria (U. LP), in an acute (4 h) cyclophosphamide (CYP)-induced cystitis female mouse model. Representative **(A–H)** images, **(I)** upper LP and **(K)** deeper LP fluorescence intensity curves of pAKT immunoreactivity (CY3, red) in the urinary bladder (10 μm sections) in female mice with **(E)** acute (4 h) cyclophosphamide (CYP)-induced cystitis (200 mg/kg, i.p.) and **(C,D)** controls (no CYP), **(F)** pre-treated with imatinib mesylate via oral gavage (250 mg/kg, 5 days, 1X/day). **(A,B,G,H)** Bladder tissue was co-stained for pPDGFRα (FITC, green), and the corresponding images are displayed to provide orientation. 4 h CYP treatment significantly (*p* ≤ 0.05) increased pAKT expression in the **(J)** U. LP (1.4-fold) and the **(L)** D. LP (1.2-fold), compared to vehicle controls. Imatinib prevention significantly (*p* ≤ 0.001) reduced pAKT expression in the **(L)** D. LP (2.2-fold), but not the **(J)** U. LP in female mice with 4 h CYP-induced cystitis. Raw IHC values (intensity units, 8-bit) are presented as fold change values ± SEM with respect to the control group (vehicle, no CYP). *n* = 4–6. *p ≤ 0.05; **p ≤ 0.01; ***p ≤ 0.001; ****p ≤ 0.0001 by two-way ANOVA with Tukey’s multiple comparisons test. Tissues were processed simultaneously. Microscope and camera settings were kept constant across all groups. Fluorescence intensity was measured using Volumetry image analysis software (VolumetryG9a, Grant Hennig). 10 μm sections. Scale bar = 50 μm. U, urothelium; U. LP, upper lamina propria; D. LP, deeper lamina propria; IU, intensity units.

**FIGURE 9:**
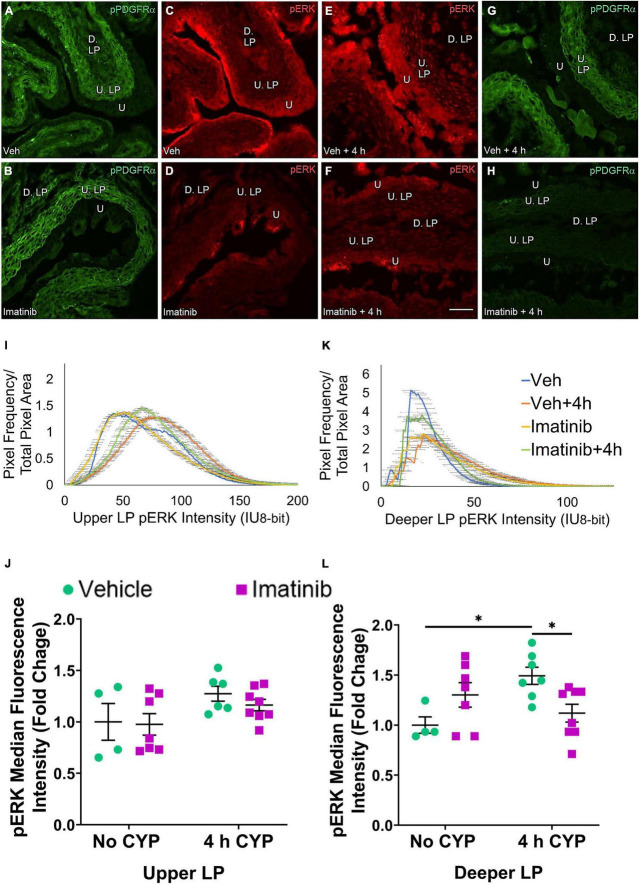
Transurethral bladder infusion of imatinib mesylate reduces pERK expression in the deeper lamina propria (D. LP), but not upper lamina propria (U. LP), in an acute (4 h) cyclophosphamide (CYP)-induced cystitis female mouse model. Representative **(A–H)** images, **(I)** upper LP and **(K)** deeper LP fluorescence intensity curves of pERK immunoreactivity (CY3, red) in the urinary bladder (10 μm sections) in female mice with **(E)** acute (4 h) cyclophosphamide (CYP)-induced cystitis (200 mg/kg, i.p.) and **(C,D)** controls (no CYP), **(F)** treated with imatinib mesylate via transurethral intravesical infusion (50 μM, 30 min). **(A,B,G,H)** Bladder tissue was co-stained for pPDGFRα (FITC, green), and the corresponding images are displayed to provide orientation. 4 h CYP treatment significantly (*p* ≤ 0.05) increased pERK expression in the **(L)** D. LP (1.5-fold) but not the **(J)** U. LP, compared to vehicle controls. Imatinib treatment significantly (*p* ≤ 0.05) reduced pERK expression in the **(L)** D. LP (1.3-fold), but not the **(J)** U. LP in female mice with 4 h CYP-induced cystitis. Raw IHC values (intensity units, 8-bit) are presented as fold change values ± SEM with respect to the control group (vehicle, no CYP). *n* = 4–8. *p ≤ 0.05; **p ≤ 0.01; ***p ≤ 0.001; ****p ≤ 0.0001 by two-way ANOVA with Tukey’s multiple comparisons test. Tissues were processed simultaneously. Microscope and camera settings were kept constant across all groups. Fluorescence intensity was measured using Volumetry image analysis software (VolumetryG9a, Grant Hennig). Scale bar = 50 μm. U, urothelium; U. LP, upper lamina propria; D. LP, deeper lamina propria; IU, intensity units.

**FIGURE 10:**
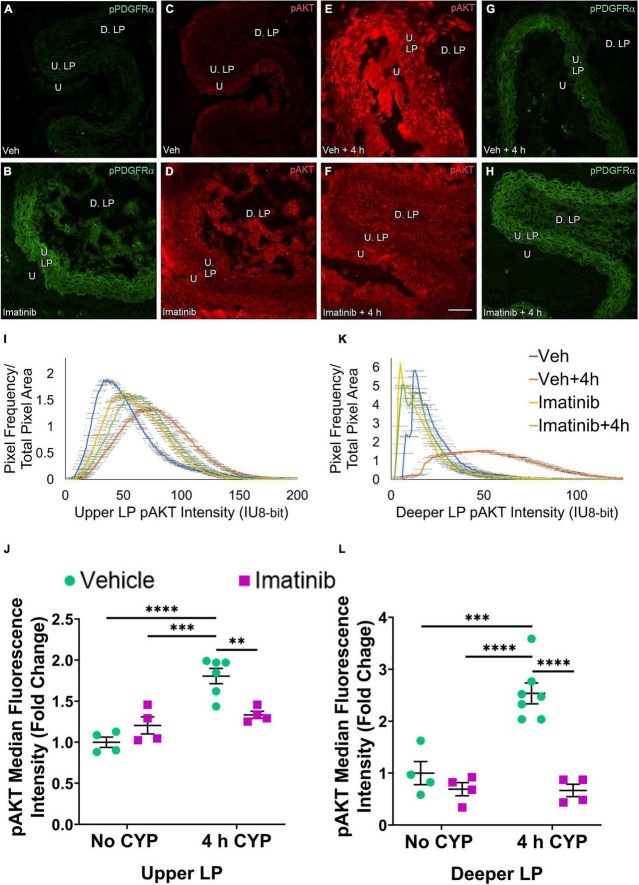
Transurethral bladder infusion of imatinib mesylate reduces pAKT expression in the upper LP (U. LP) and deeper lamina propria (D. LP), in an acute (4 h) cyclophosphamide (CYP)-induced cystitis female mouse model. Representative **(A–H)** images, **(I)** upper LP and **(K)** deeper LP fluorescence intensity curves of pAKT immunoreactivity (CY3, red) in the urinary bladder (10 μm sections) in female mice with **(E)** acute (4 h) cyclophosphamide (CYP)-induced cystitis (200 mg/kg, i.p.) and **(C,D)** controls (no CYP), **(F)** treated with imatinib mesylate via transurethral intravesical infusion (50 μM, 30 min). **(A,B,G,H)** Bladder tissue was co-stained for pPDGFRα (FITC, green), and the corresponding images are displayed to provide orientation. 4 h CYP treatment significantly (0.0001 ≤ *p* ≤ 0.001) increased pAKT expression in the **(J)** U. LP (1.8-fold) and the **(L)** D. LP (2.5-fold), compared to vehicle controls. Imatinib treatment significantly (0.0001 ≤ *p* ≤ 0.01) reduced pAKT expression in the **(J)** U. LP (1.4-fold) and the **(L)** D. LP (3.8-fold) in female mice with 4 h CYP-induced cystitis. Raw IHC values (intensity units, 8-bit) were converted to and are displayed as fold change values ± SEM with respect to the control group (vehicle, no CYP). *n* = 4–7. *p ≤ 0.05; **p ≤ 0.01; ***p ≤ 0.001; ****p ≤ 0.0001 by two-way ANOVA with Tukey’s multiple comparisons test. Tissues were processed simultaneously. Microscope and camera settings were kept constant across all groups. Fluorescence intensity was measured using Volumetry image analysis software (VolumetryG9a, Grant Hennig). Scale bar = 50 μm. U, urothelium; U. LP, upper lamina propria; D. LP, deeper lamina propria; IU, intensity units.

Two regions of the LP were analyzed: the U. LP, directly underneath the urothelium, sometimes called the suburothelium and the D. LP, defined as the loosely distributed tissue between the U. LP and detrusor (Figures 7–10). Separate cohorts of mice were used in the following studies.

#### Upper LP

##### Prevention (Gavage), pERK

In the prevention studies (gavage, 250 mg/kg, 5 days, 1X/day), pERK U. LP expression is low in the vehicle controls and is dramatically increased in the vehicle pre-treated, 4 h CYP condition (Figures 7C,E). Imatinib control and imatinib pre-treated, 4 h CYP pERK expression is greater than vehicle, but not to the same extent as the vehicle pre-treated, 4 h CYP condition (Figures 7C–F). All groups display bell-shaped pERK intensity curves in the U. LP (Figure 7I). Most of the pixels within the U. LP exhibit a peak fluorescence intensity (25–50 intensity unit; IU) for the vehicle, imatinib, and imatinib prevention with 4 h CYP groups. The vehicle pre-treated, 4 h CYP group is also bell-shaped with a peak, but exhibits broader fluorescence across a range of intensities (10–150 IU), producing a flatter curve. The vehicle pre-treated, 4 h CYP group exhibits a rightward shift, with more pixels fluorescing at greater intensities. Imatinib control and imatinib pre-treated, 4 h CYP groups also demonstrate rightward shifts compared to vehicle control, but lesser than the vehicle, 4 h CYP group (Figure 7I).

Acute (4 h) CYP treatment (200 mg/kg, i.p.) significantly (*p* ≤ 0.001) increased pERK fluorescence intensity in the U. LP of female mice compared to vehicle and imatinib controls (1.5–1.9-fold) (Figure 7J). Imatinib prevention via oral gavage significantly (*p* ≤ 0.01) (1.5-fold) reduced pERK fluorescence intensity in the U. LP in mice with acute (4 h) CYP-induced cystitis (Figure 7J).

##### Prevention (Gavage), pAKT

pAKT expression is increased in the U. LP in mice 4 h CYP-induced cystitis, pre-treated with vehicle, compared to the imatinib and vehicle controls, and imatinib pre-treated 4 h CYP condition (Figures 8C–F). Like pERK expression in the U. LP, pAKT expression formed bell-shaped, high peak curves (Figure 8I). The vehicle pre-treated, 4 h CYP group exhibits a small rightward shift (i.e., brighter intensities) (10–150 IU) away from vehicle (10–100 IU). This shift is not different from the imatinib control and imatinib pre-treated, 4 h CYP groups with many pixels from these groups falling within the same fluorescence intensity range (Figure 8I).

Four hour CYP treatment significantly (*p* ≤ 0.05) (1.4-fold) increased pAKT fluorescence intensity in the U. LP of female mice compared to vehicle control (Figure 8J). Imatinib prevention via oral gavage did not affect pAKT fluorescence in the U. LP in mice with 4 h CYP-induced cystitis (Figure 8J).

##### Treatment (Intravesical Infusion), pERK

pERK expression is not different in the U. LP in mice treated with imatinib or vehicle via intravesical infusion, with or without 4 h CYP (Figures 9C–F). In the U. LP, all groups exhibit a wide range of fluorescence intensities, producing wide, flat curves. Although wide, each group exhibits a peak intensity level (50, 60, 75 IU). Most pixels overlap within similar intensity ranges for all groups (Figure 9I).

Upper LP pERK fluorescence intensity was not significantly different in mice with 4 h CYP-induced cystitis that received imatinib or saline intrabladder infusions, compared to saline infused controls (Figure 9J). Imatinib intrabladder infusion also did not significantly affect U. LP pERK fluorescence in mice with 4 h CYP-induced cystitis (Figure 9J).

##### Treatment (Intravesical Infusion), pAKT

pAKT expression in the U. LP in mice treated with 4 h CYP is increased compared to the vehicle condition (Figures 10C,E). Imatinib control and imatinib treated groups exhibit greater pAKT expression than the vehicle control group, but less than the 4 h CYP, vehicle treated group (Figures 10C–F). These groups display bell-shaped, high peak pAKT intensity curves (Figure 10I). The vehicle treated, 4 h CYP group exhibits a flatter curve and rightward shift (i.e., brighter intensities) (20–150 IU). Imatinib control and imatinib treated, 4 h CYP groups also demonstrate rightward shifts (20–125 IU) compared to vehicle control, but less than the vehicle, 4 h CYP group (10–125 IU) (Figure 10I).

Four hour CYP treatment (200 mg/kg, i.p.) significantly (*p* ≤ 0.001) increased pAKT fluorescence intensity in the U. LP of female mice compared to vehicle and imatinib controls (1.5–1.8-fold) (Figure 10J). Intravesical imatinib treatment significantly (*p* ≤ 0.01) (1.4-fold) reduced pAKT fluorescence intensity in mice with 4 h CYP-induced cystitis (Figure 10J).

#### Deeper LP

##### Prevention (Gavage), pERK

pERK expression in the D. LP was reduced in the imatinib pre-treated, 4 h CYP group compared to the vehicle pre-treated, 4 h CYP group (Figures 7E,F). Unlike the U. LP, the D. LP produced variable rising intensity curves, possibly due to the more diffuse organization of D. LP tissue. Pixel intensities from each group largely overlapped, but imatinib control and imatinib pre-treated, 4 h CYP groups displayed more pixels at lower (<25 IU) intensities compared to the vehicle pre-treated, 4 h CYP group (25–100 IU) (Figure 7K). Imatinib prevention significantly (*p* ≤ 0.05) (1.6-fold) reduced pERK fluorescence intensity in mice with 4 h CYP-induced cystitis (Figure 7L).

##### Prevention (Gavage), pAKT

pAKT expression in the D. LP is greater with 4 h CYP treatment than controls (Figures 8C–E). Imatinib prevention reduces pAKT expression in the 4 h CYP condition (Figures 8E,F). The imatinib control and vehicle pre-treated, 4 h CYP groups displayed many pixels fluorescing across a broad range, producing very wide, flat curves (20–100 IU). The vehicle control and imatinib pre-treated, 4 h CYP groups displayed sharper peaks, with most pixels fluorescing at lower (<25 IU) intensity levels (Figure 8K).

Four hour CYP treatment significantly (*p* ≤ 0.05) (1.5-fold) increased pAKT fluorescence intensity in the D. LP that was significantly (*p* ≤ 0.001) (2.2-fold) reduced by imatinib prevention via gavage (Figure 8L).

##### Treatment (Intravesical Infusion), pERK

pERK expression in the D. LP is greater in the vehicle treated, 4 h CYP group compared to vehicle control (Figures 9C,E). Imatinib treatment appears to reduce pERK expression in the 4 h CYP condition (Figures 9E,F). These groups exhibit peaked, variable intensity curves. The imatinib controls and vehicle treated, 4 h CYP group display a rightward shift compared to vehicle controls and the vehicle treated, 4 h CYP group (Figure 9K).

Four hour CYP treatment significantly (*p* ≤ 0.05) (1.5-fold) increased pERK fluorescence intensity in the D. LP that was significantly (*p* ≤ 0.05) (1.3-fold) reduced by imatinib treatment via intravesical infusion (Figure 9L).

##### Treatment (Intravesical Infusion), pAKT

pAKT expression in the D. LP is greater with 4 h CYP treatment compared to vehicle control (Figures 10C–E). Imatinib treatment reduces expression comparable to control levels in the 4 h CYP condition (Figures 10C–F). Most pixels in the vehicle control, imatinib control and imatinib treated, 4 h CYP groups coalesced around low (<25 IU) intensity levels, producing sharp, high peaks. The vehicle treated, 4 h CYP group demonstrated a dramatic rightward shift (i.e., brighter intensities), with many pixels fluorescing across a broad range (25–100 IU) (Figure 10K).

Four hour CYP treatment significantly (*p* ≤ 0.001) (2.5-fold) increased pAKT fluorescence intensity in the D. LP that was significantly (*p* ≤ 0.0001) (3.8-fold) reduced by imatinib treatment via intravesical infusion (Figure 10L).

## Discussion

We and others (Kubota et al., 2004, 2006; Biers et al., 2006; Abrams et al., 2012; Deng et al., 2013; Kjell et al., 2015; Preis et al., 2015; Sancho et al., 2017; Giglio et al., 2018) have previously demonstrated LUT improvements with imatinib administration in animal models of bladder dysfunction. These studies expand upon previous reports (Abrams et al., 2012; Kjell et al., 2015) examining the effect of imatinib on inflammatory mediators in the serum, spinal cord, and peripheral lymphoid organs (i.e., spleen, bone marrow, thymus) in rats with contusion SCI. However, the effect of imatinib on the urinary bladder inflammatory milieu and signaling kinases has not been previously investigated in IC/BPS animal models. Our studies demonstrate: (1) imatinib prevention via oral gavage (i.e., 250 mg/kg, 1X/day, 5 days), but not imatinib treatment via a single intrabladder infusion (i.e., 50 μM, 30 min), reduces urinary bladder inflammatory mediator (e.g., VEGF, BDNF, CCL2, IL-6) mRNA and protein expression, and (2) both imatinib prevention and treatment designs reduce kinase (pERK, pAKT) immunoreactivity (IR) in the bladder LP in female mice with acute (4 h) CYP-induced cystitis.

Imatinib and other PDGFRα inhibitors have been used in a wide variety of model systems and clinical studies (e.g., SCI, stroke, MS, retinal neovascularization, fibrosis, CML, GI stromal tumors, glioblastoma) to address effects on the immune response (Buchdunger et al., 2002; Ma et al., 2011; Hayashi et al., 2012; Adzemovic et al., 2013; Kjell et al., 2015; Roskoski, 2018; Cohen et al., 2021). Here, we examine inflammatory mediators (e.g., NGF, BDNF, VEGF, CCL2, IL-6) previously shown to increase expression and exhibit functional effects in micturition pathways in rodent models of CYP-induced cystitis (Vizzard, 2000b; Malley and Vizzard, 2002; Hu et al., 2005; Zvara and Vizzard, 2007; Cheppudira et al., 2008; Girard et al., 2011a,^2016^; Arms et al., 2013; Tooke et al., 2019). Prevention by oral gavage of imatinib reduced VEGFaa, VEGFab, BDNF, IL-6 and CCL2 mRNA, and VEGF and IL-6 protein expression in the LP and whole bladder, respectively, in mice with acute CYP-induced cystitis. However, imatinib did not affect NGF LP mRNA expression. Treatment with imatinib by intravesical infusion in mice with acute CYP-induced cystitis did not significantly affect mRNA or protein expression of the inflammatory mediators examined.

Previously, we demonstrated bladder functional improvements (i.e., decreased voiding frequency), with imatinib in a (1) prevention design (before CYP) by oral gavage, or (2) treatment (after CYP) by transurethral intrabladder infusion in mice with 4 h CYP-induced cystitis (Perkins et al., 2022). Imatinib may have affected these inflammatory mediators with the prevention, but not treatment, design due to the frequency and duration of imatinib administration. The pharmacokinetics of imatinib and previous studies (Buchdunger et al., 2002; Peng et al., 2005; Abrams et al., 2012; Kjell et al., 2015; Sancho et al., 2017) suggest daily, repeated dosing of imatinib like our prevention design protocol (1X/day for 5 days) is effective in contrast to a single treatment (i.e., single intrabladder infusion). Our differential results may also be due to the delivery route of imatinib (systemic vs. intrabladder). In addition to PDGFRα, imatinib also inhibits the tyrosine kinases PDGFRβ, c-KIT, and ABL (Buchdunger et al., 1996, 2000; Capdeville et al., 2002). These tyrosine kinases are highly conserved across species and widely expressed throughout the body on various cell types (Andrae et al., 2008; Colicelli, 2010; Lennartsson and Rönnstrand, 2012). With the prevention design, imatinib was systemically delivered allowing the drug to act at bladder and non-bladder sites with pervasive receptor expression.

When imatinib was delivered via intravesical infusion, improvements in bladder function in CYP-treated mice were demonstrated within 30 min and persisted during the entire recording period (Perkins et al., 2022). In the present studies, no changes in the inflammatory mediators examined were detected following intravesical imatinib infusion in mice with acute CYP-induced cystitis. It is possible that intravesical effects of imatinib on the inflammatory milieu may require more time to be detected and/or imatinib may affect other inflammatory mediators that were not evaluated in these studies. Additional time points and mediators should be examined. However, changes in the inflammatory mediators that we evaluated in this study are unlikely to underlie the early bladder functional improvements in mice with 4 h CYP-induced cystitis and other mechanisms of action for imatinib should be considered.

Numerous reports in a variety of models (e.g., SCI, stroke, MS) suggest that imatinib alters endothelial tight junction protein expression (e.g., claudin 5, occludin, *e*-cadherin, connexin 43, beta-catenin) and improves vascular integrity (Vrekoussis et al., 2006; Su et al., 2008; Ma et al., 2011; Abrams et al., 2012; Aman et al., 2012; Adzemovic et al., 2013; Chislock and Pendergast, 2013; Zhan et al., 2015; Langer et al., 2019; Wang et al., 2020). Tight junction proteins (e.g., occludins, claudins) are also expressed by urothelial cells and contribute to the exceptionally high resistance of the urothelium (Rickard et al., 2008; Birder and Andersson, 2013; Chung et al., 2014). Intravesical imatinib may mediate functional improvements by altering urothelial barrier characteristics (e.g., tight junction, uroplakin, glycosaminoglycan protein expression). Future studies should examine effects of imatinib on urothelium tight junction protein expression and transepithelial resistance in control mice and in mice with CYP-induced cystitis. In addition, when considering the effects of imatinib delivered intravesically, one should consider the depth of penetration of intravesically infused substances and cell types affected. Interstitial cells exhibit heterogeneous morphology and exhibit differential receptor and channel expression, properties, and function according to tissue distribution in the LP (upper vs. lower) and tissue (LP vs. detrusor) (Gevaert et al., 2014b; Neuhaus et al., 2018; Steiner et al., 2018). Thus, effects of intravesical imatinib treatment may be affected by frequency and duration of imatinib treatment and populations of interstitial cells affected.

Increased NGF and BDNF expression in the urinary bladder with CYP-induced cystitis has been demonstrated in preclinical animal models and human patients with IC/BPS (Vizzard, 2000b; Girard et al., 2016; Coelho et al., 2019). Unexpectedly in the present study, NGF mRNA and BDNF mRNA and protein expression were not significantly different in mice with 4 h CYP-induced cystitis that subsequently received transurethral intravesical saline infusion compared to controls (no CYP, intravesical saline infusion). The lack of effect of CYP on NGF and BDNF mRNA may be partially due to solely examining the LP, without the urothelium or detrusor as previously demonstrated.

We previously demonstrated pERK and pAKT functional involvement and expression in the micturition system in CYP-induced cystitis (Corrow and Vizzard, 2007, 2009; Arms and Vizzard, 2011). The MAPK/ERK and PI3K/AKT pathways regulate many processes including cell growth, proliferation, migration, survival, angiogenesis, cell death, inflammatory mediator expression and secretion (Brazil and Hemmings, 2001; Miranda et al., 2005; Li et al., 2006; Hers et al., 2011; Fernandes et al., 2016; Zegeye et al., 2018; Lavoie et al., 2020; Sharma et al., 2020). In rodent models of CYP-induced cystitis, functional and pharmacological studies suggest pERK and pAKT may induce pCREB, mTOR, type 1 collagen, and cytokine expression, including IL-6 (Kay et al., 2013; Qiao et al., 2014; Sun et al., 2015). In our studies, the reduction in kinase expression with imatinib was most prominent with pAKT and in the D. LP. A major downstream target of pAKT is mTOR (Manning and Toker, 2017) which may contribute to IC/BPS (Yang et al., 2012; Lin et al., 2015; Liang et al., 2016; Xie et al., 2018; Liu et al., 2020). Intrathecal blockade of the PI3K/AKT-mTOR pathway reduced bladder hyperactivity, somatic sensitivity, and spinal cord substance P and CGRP protein expression in female rats with chronic CYP-induced cystitis (Liang et al., 2016). Future studies concerning signaling mechanisms of PDGFRα + interstitial cells and their contribution to cystitis should examine downstream kinase targets including mTOR.

In conclusion, our studies suggest that imatinib may affect the urinary bladder inflammatory milieu and signaling kinases differently, depending on delivery route, frequency, and duration of treatment. Imatinib is likely mediating effects through multiple mechanisms (e.g., reducing inflammatory mediators, enhancing endothelial and urothelial barriers) which may be dependent on frequency of delivery and delivery route. Imatinib prevention via gavage may be improving functional urinary outcomes, in part, by affecting the bladder inflammatory milieu in female mice with acute CYP-induced cystitis. Imatinib treatment via intravesical infusion in CYP-induced cystitis did not affect urinary inflammatory mediator expression at the examined time point but could be utilizing other mechanisms to mediate functional improvements including altering urothelial barrier properties. Additionally, PDGFRα + interstitial cell signaling may be mediated by both pERK and pAKT pathways in cystitis. Future studies should examine the effect of imatinib on urothelial barrier function and downstream kinase targets (e.g., mTOR), to further elucidate the mechanism(s) by which imatinib improves urinary bladder function in CYP-induced cystitis.

## Data Availability Statement

The original contributions presented in the study are included in the article/supplementary material, further inquiries can be directed to the corresponding author/s.

## Ethics Statement

The animal study was reviewed and approved by the University of Vermont Institutional Animal Care and Use Committee.

## Author Contributions

MP, BG, and MV conceived and designed the research, interpreted the results of experiments, edited and revised the manuscript. MP, SC, and BG performed the experiments. MP and GH analyzed the data. GH instructed MP in proper use of Volumetry software (G9a). MP prepared the figures and drafted the manuscript. MP, BG, SC, GH, and MV approved final version of manuscript. All the authors contributed to the article and approved the submitted version.

## Author Disclaimer

The National Institutes of Health had no role in the experiments described, including the design, data collection, and analysis of studies performed in the Vizzard laboratory, decision to publish, or preparation of the manuscript. The contents are solely the responsibility of the authors and do not necessarily represent the official views of the National Institutes of Health.

## Conflict of Interest

The authors declare that the research was conducted in the absence of any commercial or financial relationships that could be construed as a potential conflict of interest.

## Publisher’s Note

All claims expressed in this article are solely those of the authors and do not necessarily represent those of their affiliated organizations, or those of the publisher, the editors and the reviewers. Any product that may be evaluated in this article, or claim that may be made by its manufacturer, is not guaranteed or endorsed by the publisher.

## Footnotes

1
www.graphpad.com

## Abbreviations

IC/BPS: interstitial cystitis/bladder pain syndrome
OAB: overactive bladder
SCI: spinal cord injury
LUT: lower urinary tract
LP: lamina propria
U. LP: upper lamina propria
D. LP: deeper lamina propria
DRG: dorsal root ganglia
WT: wild type
PDGFRα: platelet-derived growth factor receptor alpha
PDGFRβ: platelet-derived growth factor receptor beta
c-KIT: tyrosine-protein kinase KIT
BCR-ABL: breakpoint cluster region-Abelson murine leukemia
TrpV4: transient receptor potential vanilloid-type 4 (Trpv4)
NGF: nerve growth factor
BDNF: brain-derived neurotrophic factor
VEGF: vascular endothelial growth factor
IL-6: interleukin-6
CCL2: C-C motif chemokine ligand 2
MAPK/ERK: mitogen-activated protein kinase/extracellular signal-related kinase
PI3K/AKT: phosphatidylinositol 3-kinase/protein kinase B
p: phosphorylated
IMI: intermicturition interval
IV: infused volume
CYP: cyclophosphamide
i.p.: intraperitoneal
s.c.: subcutaneous
h: hour(s)
min: minutes
ANOVA: analysis of variance
qRT-PCR: quantitative reverse transcription-polymerase chain reaction
ELISA(s): enzyme-linked immunosorbent assay(s)
IHC: immunohistochemistry
IU: intensity units.
